# TCA Cycle Defects and Cancer: When Metabolism Tunes Redox State

**DOI:** 10.1155/2012/161837

**Published:** 2012-07-19

**Authors:** Simone Cardaci, Maria Rosa Ciriolo

**Affiliations:** ^1^Department of Biology, University of Rome “Tor Vergata”, Via della Ricerca Scientifica, 00133 Rome, Italy; ^2^IRCCS San Raffaele Pisana, Via di Val Cannuta, 00166 Rome, Italy

## Abstract

Inborn defects of the tricarboxylic acid (TCA) cycle enzymes have been known for more than twenty years. Until recently, only recessive mutations were described which, although resulted in severe multisystem syndromes, did not predispose to cancer onset. In the last ten years, a causal role in carcinogenesis has been documented for inherited and acquired alterations in three TCA cycle enzymes, succinate dehydrogenase (SDH), fumarate hydratase (FH), and isocitrate dehydrogenase (IDH), pointing towards metabolic alterations as the underlying hallmark of cancer. This paper summarizes the neoplastic alterations of the TCA cycle enzymes focusing on the generation of pseudohypoxic phenotype and the alteration of epigenetic homeostasis as the main tumor-promoting effects of the TCA cycle affecting defects. Moreover, we debate on the ability of these mutations to affect cellular redox state and to promote carcinogenesis by impacting on redox biology.

## 1. Introduction

Cancer cells differ from normal ones due to a plethora of oncogenes-driven biochemical changes designed to sustain an high rate of growth and proliferation [1]. The first tumor-specific alteration in metabolism was reported at the beginning of the 20th century by Warburg [2]. His observations demonstrated that cancer cell metabolism relies on an increased glycolytic flux maintained even in the presence of oxygen (“aerobic glycolysis” or “Warburg effect”), without an associated increase in oxidative phosphorylation rate. The switch from respiration to glycolysis has usually been considered a consequence, rather than a cause, of cancer. However, in the last decade, the discovery that inherited and acquired alterations in some enzymes of tricarboxylic acid (TCA) cycle have a causal role in carcinogenesis has changed this viewpoint, pointing towards altered metabolism as the underlying hallmark of neoplastic transformation. These alterations consist of germline defects in genes encoding subunits of SDH and FH, as well as somatic mutations in coding sequence for IDH. Together with metabolomics studies documenting the alteration of HIF-dependent signaling pathway and epigenetic dynamics as main tumor-promoting effects of these mutations, a mounting body of evidence also supports how alterations in the TCA cycle enzymes may favor tumorigenesis by impacting on cellular redox state. Therefore, in this paper, we summarize the prooncogenic defects in the TCA cycle enzymes discussing their involvement in the tuning of redox environment and the engagement of redox-dependent tumorigenic signaling.

## 2. Fundamentals of the TCA Cycle

**Figure 1:**
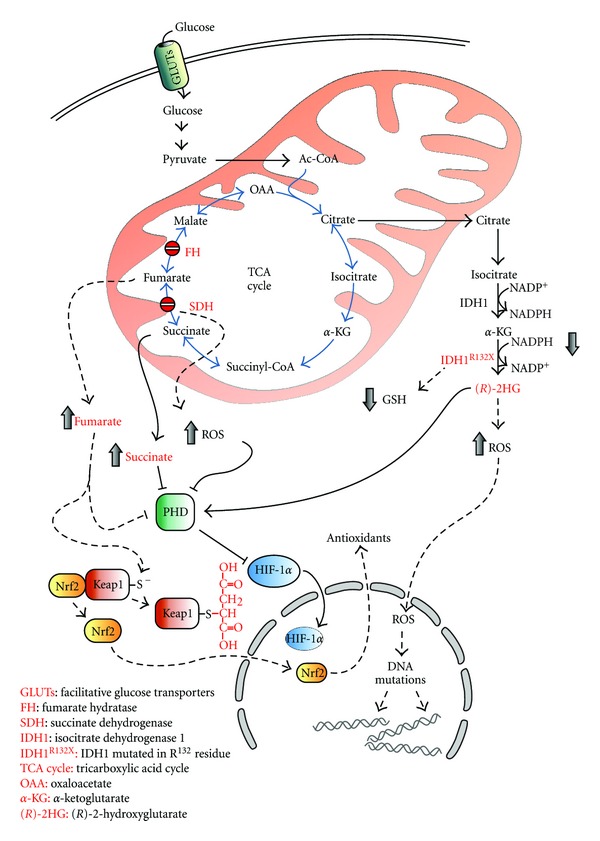
Redox alterations induced by TCA cycle defects. Redox alterations induced by mutations in SDH, FH, and IDH are shown. Loss of function of SDH increases ROS levels leading to DNA mutations and HIF-1*α* stabilization. IDH1 and IDH2 (not shown) mutations decrease GSH and NADPH levels. (*R*)-2-HG, produced by oncogenic mutations in IDH1 and IDH2, triggers ROS accumulation. Defects in FH stimulate nuclear translocation of Nrf2 and the transcription of antioxidant enzymes through the succination of Keap1. Enzymes and metabolites involved in tumor formation and redox alterations are in red. Blue arrows indicate TCA cycle reactions. Dotted arrows indicate pathways modulating cell redox state.

The TCA cycle is a core pathway for the metabolism of sugars, lipids, and amino acids [3]. It is usually presented in a naive perspective of a cyclic mitochondrial route constantly oxidizing the acetyl moiety of acetyl-coenzyme A to CO_2_, generating NADH and FADH_2_, whose electrons fuel the mitochondrial respiratory chain for ATP generation. The TCA cycle begins with the condensation of acetyl-CoA with oxaloacetate to form citrate, catalyzed by citrate synthase. Citrate can be exported to the cytoplasm, where it is used as precursor for lipid biosynthesis or remains in the mitochondria, where it is converted to isocitrate by aconitase. In the next step, *α*-ketoglutarate (*α*-KG), formed by the oxidative decarboxylation of isocitrate catalyzed by IDH, is converted to succinyl-CoA by a further decarboxylation by the *α*-KG dehydrogenase complex. Succinyl-CoA is then transformed to succinate by the succinyl-CoA synthetase. Fumarate, produced by succinate oxidation catalyzed by the SDH complex, is hydrated to malate by FH. Oxidation of malate, catalyzed by malate dehydrogenase, finally regenerates oxaloacetate, thus ensuring the completion of the cycle (Figure 1). On the mere biochemical viewpoint, the TCA cycle in nontumor cells has been divided into two stages: (i) decarboxylating, in which citrate is converted to succinyl-CoA releasing two CO_2_ molecules; (ii) reductive, which comprises the successive oxidations of succinate to oxaloacetate. Interestingly, emerging findings from the last year support the hypothesis that, in several cell systems such as (i) cancer cells containing mutations in complex I or complex III of the electron transport chain (ETC), (ii) patient-derived renal carcinoma cells with mutations in FH, (iii) cells with normal mitochondria subjected to acute pharmacological ETC, inhibition, as well as (iv) tumor cells exposed to hypoxia, the first stage of the cycle can proceed in the opposite direction through the reductive carboxylation of *α*-KG to form citrate. This allows cells to produce acetyl-coenzyme A to support *de novo* lipogenesis and their viability [4–6]. Although in physiological and resting conditions mitochondria are necessary and sufficient to perform the cycle, isoforms of some of its enzymes have been also found in the cytosol. This ensures a dual compartmentalization (cytosolic and mitochondrial) of reactions and metabolites which, being free to diffuse through the outer and the inner mitochondrial membranes by channels and active carriers, respectively, allows the cycle to respond to environmental and developmental signals, thus sustaining anabolic reactions as well as fueling the ATP-producing machinery. The TCA cycle is also a major pathway for interconversion of metabolites arising from transamination and deamination of amino acids and provides the substrates for amino acids synthesis by transamination, as well as for gluconeogenesis and fatty acid synthesis. Regulation of the TCA cycle depends primarily on a supply of oxidized cofactors: in tissues where its primary role is energy production, a respiratory control mediated by respiratory chain and oxidative phosphorylation is operative. This activity relies on availability of NAD^+^ and ADP, which in turn depends on the rate of utilization of ATP in chemical and physical work.

## 3. Genetic Defects in the TCA Cycle

Genetic defects affecting the TCA cycle enzymes have been known for more than two decades. Until recently, only recessive mutations were documented whose clinical consequences were similar to alterations in the electron transport chain (ETC) and oxidative phosphorylation [7]. These defects were associated with multisystem disorders and severe neurological damage, but no cancer predisposition, as a result of very considerably impaired ATP formation in the central nervous system. In the last ten years, dominant defects associated with oncogenesis were described in cytoplasmic and mitochondrial isoforms of three nuclear-encoded enzymes, SDH, FH, and IDH, allowing to investigate the extrametabolic roles of the TCA cycle metabolites and their signaling to tumor formation.

### 3.1. Succinate Dehydrogenase

The SDH complex (also known as succinate:ubiquinone oxidoreductase or mitochondrial complex II) is a highly conserved heterotetrameric tumor suppressor, composed by two catalytic subunits (SDHA and SDHB), which protrude into the mitochondrial matrix, and two hydrophobic subunits (SDHC and SDHD), which anchor the catalytic components to the inner mitochondrial membrane and provide the binding site for the ubiquinone, as well [8]. All the subunits are encoded by nuclear genome and, unlike most of the TCA cycle enzymes, have no cytosolic counterparts. SDH catalyzes the oxidation of succinate to fumarate in the TCA cycle with the simultaneously reduction of ubiquinone to ubiquinol in the ETC. A decade ago, mutations in SDHB, SDHC, and SDHD subunits were identified in patients with hereditary paragangliomas (hPGLs) and pheochromocytomas (PCCs), a rare neuroendocrine neoplasm of the chromaffin tissue of the adrenal medulla or derived from the parasympathetic tissue of the head and neck paraganglioma, respectively [9–12]. More recently, mutations in SDHA and the SDH assembly factor 2 (SDHAF2), required for flavination of SDH [13, 14], have been associated with hPGL/PCC syndrome [15]. The genetic defects in the SDH genes predisposing to the hPGL as well as PCC are heterozygous germline mutations, inducing the inactivation of the protein and the neoplastic transformation develops as result of loss of heterozygosity, caused by the complete loss of the enzyme function by a second mutagenic hit (usually deletion) [16]. In addition to hPGL and PCC, a number of other neoplasms have been associated with mutations in SDH genes, including gastrointestinal stromal tumors, renal cell cancers, thyroid tumors, neuroblastomas, and testicular seminoma [8].

### 3.2. Fumarate Hydratase

FH is homotetrameric TCA cycle enzyme which catalyzes the stereospecific and reversible hydration of fumarate to L-malate. Homozygous FH deficiencies result in fumaric aciduria [17], characterized by early onset of severe encephalopathy and psychomotor retardation; on the contrary, heterozygous FH mutations predispose to multiple cutaneous and uterine leiomyomas (MCUL), as well as to hereditary leiomyomatosis and renal cell cancer (HLRCC) [18, 19]. In particular, the kidney tumors in HLRCC, whose morphological spectrum include papillary type II, tubulopapilar, tubular, collecting duct, and clear cell carcinoma, are particularly aggressive. Growing evidence suggests that *FH* mutations may also be involved in the pathogenesis of breast, bladder, as well as Leydig cell tumors [20, 21]. The most common types of tumor predisposing genetic defects are missense mutations (57%), followed by frameshift and nonsense mutations (27%), as well as large-scale deletions, insertions, and duplications [22]. Like SDH, enzymatic activity of FH is completely absent in HLRCC as result of the loss of the wild-type allele in the transformed cell.

### 3.3. Isocitrate Dehydrogenase

IDH is a member of the *β*-decarboxylating dehydrogenase family of enzymes and catalyzes the oxidative decarboxylation of isocitrate to produce 2-oxoglutarate (*α*-KG) and CO_2_ in the TCA cycle. Nuclear genome encodes three isoforms of IDH: IDH1 and IDH2 are NADP^+^-dependent homodimers, whereas IDH3 is a NAD^+^-reliant heterotetrameric enzyme. Whereas IDH1 is found into cytoplasm and peroxisomes, IDH2 and IDH3 are exclusively localized into the mitochondrial matrix, and, although all three isoforms are able to decarboxylate isocitrate, IDH3 is the main form of IDH functioning in the TCA cycle under physiological conditions whereas IDH1 and IDH2 are mainly involved in the reductive glutamine metabolism, under hypoxia and ETC alterations [4, 5, 23]. Though it plays a central role in energy production, to date there have been no reports of cancer-associated mutations in any of the IDH3 subunits. Conversely, genomewide mutation analyses and high-throughput deep sequencing revealed the presence of mutations in either IDH1 or its mitochondrial counterpart IDH2 in 70% of grade II-III gliomas and secondary glioblastomas [24, 25]. Since these initial reports, mutations in IDH1 and IDH2 have been identified in 16-17% of patients with acute myeloid leukemia, in 20% of angioimmunoblastic T-cell lymphomas [26], and spotted in a variety of other malignancies at lower frequencies [27, 28] such as B-acute lymphoblastic leukemias, thyroid, colorectal, and prostate cancer [29, 30]. Unlike *SDH* and *FH* mutations in hPGL and HLRCC, respectively, IDH1 and IDH2 mutations are somatic and monoallelic. Moreover, whereas mutations in *SDH* and *FH* occur throughout the gene, the majority of IDH mutations identified in gliomas and AML are changes in the amino acid residues R132 in IDH1 and either R172 or R140 in IDH2 [31]. As result of these alterations, mutated IDHs are unable to efficiently catalyze the oxidative decarboxylation of isocitrate and acquire a neomorphic catalytic activity that allows a NADPH-dependent reduction of *α*-KG into the oncometabolite (*R*)-2-hydroxyglutaric acid ((*R*)-2HG) [31, 32].

## 4. Mechanisms of Tumorigenesis Caused by the TCA Cycle Defects

The finding that many tumors arousing from mutations in both *SDH* and *FH* genes are characterized by hypoxic features has suggested that the activation of the hypoxia-inducible transcription factor-1*α* (HIF-1*α*) could play a supportive role in the tumorigenic processes induced by TCA cycle dysfunctions. Indeed, HIF-1*α* is known to coordinate the biochemical reprogramming of cancer cells aimed to sustain their growth and proliferation as well as tumor vascularization [33–35]. The causal link between TCA cycle dysfunction and HIF-1*α* activation was initially suggested by Selak and coworkers demonstrating that the accumulation of succinate in SDH-deficient cells causes the inhibition of prolyl 4-hydroxylases (PHDs), a negative regulators of the stability of the *α* subunit of HIF [36]. The PHDs are members of the superfamily of *α*-KG-dependent hydroxylases, which couple the hydroxylation of the substrates with the oxidation of *α*-KG to succinate in reactions that are dependent on O_2_ and Fe^2+^ [37]. In normoxic conditions, PHDs hydroxylate two proline residues in the oxygen-dependent degradation domain of HIF-1*α*, allowing it to be polyubiquitinated and degraded *via* proteasome. The accumulated succinate in SDH-deficient or SDH-inactive cells impairs PHDs activity leading to HIF-1*α* stabilization under normoxic conditions (pseudohypoxia) [36]. Similarly to succinate, also fumarate, which accumulates in tumors harboring loss of FH function, has been demonstrated to be potent inhibitors of PHDs [38]. Interestingly, fumarate-mediated stabilization of HIF was observed to induce the upregulation of several HIF-target genes, including those that stimulate cell growth and angiogenesis, allowing to hypothesize pseudohypoxia response as a plausible mechanism for HLRCC onset [38]. Despite that this large body of evidence showed a direct link between HIF-1*α* expression and tumorigenesis, recent findings have raised some questions about the protumorigenic role of pseudohypoxic adaptation in all types of tumors arousing from TCA cycle defects. The first question was raised from the study of Adam and colleagues. They demonstrated that neither the presence of HIF nor the absence of PHDs is required for hyperplastic renal cysts formation (typical hallmark of HLRCC) in a kidney-specific *Fh1* (the ortholog of human FH) knockout mice that recapitulates many features of the human disease [39], suggesting that alternative oncogenic actions of fumarate could be responsible for HLRCC generation (see next paragraph). In addition to this study, depicting HIF as a sort of “bystander player” in the onset of tumors harboring FH mutations, another report indicated this transcription factor as a tumor-suppressor protein in tumors carrying IDH1/2 mutations. Indeed, as demonstrated by Koivunen and colleagues, contrarily to succinate and fumarate, (*R*)-2HG stimulates PHDs activity, driving, in such a way, HIF-1*α* for proteasome-mediated degradation [40]. Moreover, they pointed out that HIF-1*α* downregulation enhances the proliferation of human astrocytes and promotes their transformation, providing a justification for exploring PHDs inhibition as a potential treatment strategy for tumors harboring IDH1/2 mutations [40].

As member of the *α*-KG-dependent hydroxylases, PHDs catalyze the hydroxylation of a wide range of substrates, besides HIF-1*α* [37]. Therefore, the reduced hydroxylation of PHD targets may contribute to tumorigenesis regardless of HIF-1*α* activity and the acquisition of a hypoxic signature. For instance, it has been proposed that SDH deficiency could impair PHD-dependent programmed cell death of neurons, therefore setting the stage for neoplastic transformation of neuronal cells. This hypothesis finds support in the recent studies demonstrating that the proapoptotic activity of the prolyl hydroxylase EglN3 requires a functional SDH, being feedback inhibited by succinate [41, 42]. Since EglN3 is required during development to allow the programmed cell death of some sympathetic neuronal precursor cells, its inhibition, elicited by the elevation of succinate levels, could play a role in the pathogenesis of tumors arousing from a defective developmental apoptosis, such as pheochromocytomas.

Highlighting the HIF-1*α*-independent tumorigenic mechanisms, growing body of evidence clearly places the alteration of TCA flux upstream of the epigenetic dynamics as well. Histone methylation is an important epigenetic modification which has been demonstrated to regulate gene expression by modifying chromatin structure and, thereby, fine-tuning the binding of transcription factors [43, 44]. One of the most studied enzymes regulating histone methylation signature are the Jumonji C-terminal domain (JmjC) family of histone demethylases [45]. As they remove the methyl groups on the arginine and lysine residues of histones after performing an *α*-KG- and oxygen-dependent hydroxylation, they have been included in the *α*-KG-dependent hydroxylases family. It was shown that succinate accumulation, in SDH-deficient cells, negatively affects the activity of many members of such class of histone demethylase. For instance, succinate-mediated JMJD3 inhibition leads to changes in the methylation mark of histone H3 on arginine [46]. Furthermore, in a yeast model of paraganglioma, the histone demethylase, Jhd1, was found to be inhibited by succinate accumulation in an *α*-KG-competitive manner [47]. Similarly, recent studies demonstrate that, besides SDH alterations, also IDH1/2 defects are associated with hypermethylated phenotype. Indeed, in cells harboring *IDH1/2* mutations, intracellular (*R*)-2-HG levels can reach the value of 10 mM. These concentrations promote the competitive inhibition of the *α*-KG-dependent histone N^*ε*^-lysine demethylase JMJD2A, and the ten-eleven translocation (TET) family of 5-methylcytosine (5mC) hydroxylases, a class of protein mediating the *α*-KG-dependent removal of methyl mark from 5-methylcytosines, resulting in an enhanced histone and DNA methylation, respectively [48, 49]. Interestingly, although fumarate is able to inhibit HIF-regulating PHDs similarly to succinate, no evidence attesting its putative capability to mirror its cognate metabolite succinate in affecting histone methylation has been documented so far. In addition, as TET enzymes are members of the *α*-KG-dependent hydroxylases family, a putative ability of both succinate and fumarate in their inhibition can be reasonably argued. On the basis of the ability of the epigenetic alterations to affect lineage-specific differentiation and to result in the activation of oncogenes or silencing of tumor suppressors [44, 50, 51], the competitive inhibition of histone and DNA demethylases elicited by defects in fluxes of TCA cycle metabolites may drive tumorigenesis by promoting cell transformation and uncontrolled proliferation.

## 5. Redox-Dependent Tumorigenic Alterations Elicited by the TCA Cycle Defects

Apart from the mere metabolic viewpoint, compelling evidence suggests that the reactive oxygen species (ROS), produced by a deregulated mitochondrial functioning, might trigger the oncogenic signal or, at least, participate in the progression of tumors characterized by defects in the TCA cycle enzymes (Figure 1). This assumption finds support in the observation that, compared with their normal counterparts, many types of cancer cells have increased levels of ROS generated by a defective mitochondrial electron-transport chain [52–54]. By exploiting their chemical reactiveness with biomolecules, such as nucleic acids, ROS are known to induce several types of DNA damages, including depurination and depyrimidination, single- and double-stranded DNA breaks, base and sugar modifications, and DNA-protein crosslinks. In such a way, permanent modifications of DNA, resulting from sustained prooxidant conditions, drive the mutagenic events underlying carcinogenesis.

The observation that specific SDHC mutant (*mev-1*) of the *C. elegans* nematode was able to generate superoxide O_2_^•−^ [55, 56] suggested the possibility that ROS could have a causal role in the pathogenesis of tumors bearing defects in the TCA cycle. This hypothesis was further strengthened by the evidence that mouse fibroblasts transfected with a murine equivalent of the *mev-1* mutant were featured by a sustained ROS production and a significantly higher DNA mutation frequency than wild-type counterparts [57]. Although these lines of evidence supported the mutagenic role of ROS generated by defective SDH complex, no detectable DNA damages, despite an increased production of ROS and protein oxidation, was described in a *S. cerevisiae* strain lacking Sdh2 (the yeast ortholog of mammalian SDHB) [47]. To link the prooxidant state elicited by SDH dysfunctions to tumorigenesis, Guzy and colleagues proposed that the ROS could play a supportive role in the oncogenic process by contributing to the activation of HIF-1*α* [58]. Indeed, relying on a previously characterized role of respiratory chain-derived ROS as signals for HIF-1*α* stabilization under hypoxia [59, 60], it has been shown that cells expressing mutant SDHB, but not mutant SDHA, are characterized by significant mitochondrial ROS production required, together with succinate, for a complete inactivation of PHDs and HIF-1*α* stabilization [58]. Therefore, these results reinforce the role of ROS as amplifier of the pseudohypoxic response, observed in all cells carrying SDH defects, providing a biochemical rationale for the severity of SDHB mutations which are usually associated with aggressive PCC.

The capabilities of the TCA cycle defects in the tuning of cellular redox state have been supported by the evidence that also oncogenic mutations in IDH1/2 genes are associated with the oxidation of intracellular milieu. Normally, in aerobic organisms, the control of cellular redox state is ensured by the balance between the prooxidant species, mainly produced by mitochondria, NADPH oxidases or as byproduct of the intermediate metabolism, and their clearance through the synergistic action of the antioxidant enzymes and the thiol-containing antioxidants. Among the latter, the tripeptide glutathione (GSH) plays a pivotal role in determining the steady-state value of the intracellular redox potential. Indeed, its intracellular abundance (1–10 mM) allows GSH to participate, as electron donor, in the enzymatic reduction of hydrogen peroxide and lipid peroxides and in the generation of reversible *S*-glutathionylated adducts with protein thiols, preventing them to undergo irreversible forms of oxidation [61]. The capability of IDH mutations to induce oxidative intracellular conditions is linked to a decrease in GSH levels, observed both in IDH1-R132H—and IDH2-R172K—expressing glioma cells with respect to their *wt* counterparts [62]. GSH is synthesized in two ATP-dependent steps: (i) synthesis of *γ*-glutamylcysteine, from L-glutamate and cysteine *via* the rate-limiting enzyme glutamate-cysteine ligase (GCL); (ii) addition of glycine to the C-terminal of *γ*-glutamylcysteine *via* the enzyme glutathione synthetase. Intracellular glutamate, required for the first reaction of GSH biosynthesis, is mainly produced by the oxidative deamination of glutamine catalyzed by the enzyme glutaminase [63]. As IDH1/2 mutant cells are characterized by lower levels of glutamate with respect to their *wt* matching parts [62], it is possible that oncogenic defects in IDH result in impaired GSH synthesis due to a lower glutamate availability, thus phenocopying the prooxidant conditions observed in glutaminase deficient cells [64]. The dampened glutamate levels could be the result of an enhanced *α*-KG demand of IDH1/2 mutant cells allowing the biosynthesis of the oncometabolite (*R*)-2-HG. This assumption is supported by the evidence that treatment of glioma cells with (*R*)-2-HG does not deplete neither glutamate nor glutathione levels [62], suggesting that many metabolic changes observed in IDH-mutated cells are not due to the direct action of (*R*)-2-HG but a consequence of its oncogenic production. The involvement of IDH1/2 mutations in the generation of prooxidant conditions is not only related to the alteration of intracellular GSH content. Indeed, the oxidative decarboxylation of isocitrate, which is impaired in all mutants of IDH1 and IDH2 proteins, is coupled to a reduced ability to generate NADPH. Moreover, the failure to sustain intracellular NADPH production is associated with an increased NADPH oxidation, necessary to allow the reductive biosynthesis of (*R*)-2-HG [31, 32, 65]. As GSH and the thiol-based antioxidant protein thioredoxin require NADPH as a source of reducing equivalents for their own regeneration [61], the altered equilibrium of NADP^+^/NADPH elicited by IDH1/2 mutations could contribute to the shift of the intracellular redox state towards more oxidizing conditions. Although, these lines of evidence bring about the ability of mutant IDH1/2 to elicit prooxidant conditions independently on the direct action of (*R*)-2-HG on human redox metabolome, it has been proposed that this oncometabolite could contribute itself to oxidize intracellular environment. Indeed, some reports demonstrate its ability to induce oxidative damages in cerebral cortex of young rats [66] and to elicit ROS generation through the stimulation of NMDA receptor [67]. Although these findings support prooxidant capability of (*R*)-2-HG, to date no striking evidence has been provided attesting its mutagenic role.

Whereas accumulating pieces of evidence support the capability of oncogenic mutations in SDH as well as IDH genes to oxidize intracellular milieu, conflicting findings do not allow for defining a clear role of FH deficiency in cellular redox state modulation. The most convincing evidence showing the capability of FH-deficient cells to promote intracellular ROS accumulation comes from the work of Sudarshan and colleagues [68]. This study demonstrated that inactivating mutations of *FH* in an HLRCC-derived cell line result in glucose-induced NADPH oxidases-mediated generation of O_2_^•−^ and ROS-dependent HIF-1*α* stabilization. On the contrary, O'Flaherty and colleagues provided clear evidence that accumulation of fumarate, due to the absence of a functional FH, is the sole mechanism responsible for the inhibition of HIF-1*α* prolyl hydroxylation, independently on defect in mitochondrial oxidative metabolism [69]. Indeed, the complete correction of HIF-1*α* pathway activation in Fh1^−/−^ MEFs by extra-mitochondrial FH expression suggests that, at least in tumors harboring FH defects, neither impaired mitochondrial function nor the consequent dependence of energy metabolism on glycolysis contributes significantly to HIF-1*α* engagement. The most substantial pieces of evidence, depicting the elevation of fumarate levels as a condition linked to the reduction of intracellular redox state, came from two recent studies demonstrating that FH loss results in the activation of nuclear factor erythroid 2-related factor 2 (Nrf2) [39, 70], the pivotal transcription factor responsible for the induction of the antioxidant-responsive-element- (ARE-) driven genes, which codify for phase II detoxification enzymes and antioxidant proteins such as glutathione S-transferases and GCL [71]. Both studies demonstrated that reconstitution of FH-deficient cells with wild-type FH or an extra-mitochondrial FH decreased fumarate levels and restored Nrf2 regulation [39, 70]. In addition, elevation of intracellular fumarate content by a membrane-permeable fumarate ester was found sufficient to induce Nrf2 and its orchestrated antioxidant program [70]. According to the current view, in resting conditions, Nrf2 is retained in the cytoplasm through its interaction with Keap1 which prevents its nuclear translocation and rules its ubiquitin-proteasome-mediated turnover, as well. However, in the presence of electrophiles as well as during redox unbalance, Keap1 is modified at several reactive cysteine residues, resulting in Nrf2 stabilization and the activation of the protective gene expression program [71, 72]. In line with this accepted model, both groups revealed by mass spectroscopy analyses that fumarate was able to succinate several cysteine residues previously shown to be electrophile targets, including Cys^151^ and Cys^288^, thereby providing a mechanistic explanation of the fumarate-induced Nrf2 activation [39, 70]. Although ROS can promote carcinogenesis by inducing oxidative damages to DNA, a recent outstanding study demonstrates that oncogene-induced Nrf2 activation promotes tumorigenesis by lowering ROS levels and conferring a more reduced intracellular environment [73]. Therefore, on the basis of these evidence, it is possible to hypothesize that the fumarate-mediated activation of the Nrf2-antioxidant pathway might drive the oncogenic signal for tumors characterized by defects in the FH enzyme. Although this assumption has not been demonstrated yet, the observation that heme oxygenase 1, one of the best defined target genes of Nrf2, is upregulated in FH-deficient cells allowing their survival [74] supports the putative causal role of Keap1 succination in the onset of tumors carrying FH defects. Furthermore, mounting bodies of evidence show that Nrf2 and its downstream genes are overexpressed in many cancer cell lines and human cancer tissues conferring them advantage for survival and growth as well as acquired chemoresistance [75, 76]. Therefore, it is possible to speculate that besides driving renal tumorigenesis, fumarate-induced succination of Nrf2 could contribute to the reduced sensitivity of particularly aggressive and recurrent forms of kidney cancer, such as HLRCC [77], to many chemotherapeutic approaches. The enhanced activation of Nrf2 observed both by Pollard and Furge groups contributes to explain the results obtained by Raimundo and coworkers in nontumor cells [78]. Indeed, they documented that FH-deficient diploid human fibroblasts are characterized by a highly reduced redox state with increased GSH levels, as result of increased expression of the GSH biosynthetic enzyme GCL. As highly reducing environment has been shown to stimulate cell proliferation [79], it is possible to hypothesize that the reduced redox state elicited by FH mutations could favor the doublings of stem-cell-like populations promoting thus the initial event of tumor formation. This assumption finds support in the observation that lower ROS levels have been found in many cancer stem cells with respect to the nontumorigenic counterparts, allowing them to maintain a high proliferative status and to prevent their differentiation [80].

## 6. Concluding Remarks

The direct involvement of TCA cycle enzymes in tumor formation has been arousing from a decade. In tumors associated with defects of SDH, FH, and IDH enzymes, the underlying mechanisms of tumorigenesis involve the accumulation of metabolites (succinate, fumarate, and (*R*)-2-HG) that convey oncogenic signals (oncometabolites). Large amount of evidence points towards the generation of pseudohypoxic phenotype and the alteration of epigenetic homeostasis as the main cancer-promoting effects of the TCA cycle affecting mutations. Besides inhibiting the *α*-KG-dependent hydroxylases, mounting body of evidence supports the ability of these oncometabolites to alter cellular redox state in precancerous as well as transformed cells. Therefore, alternatively or concomitantly to the generation of pseudohypoxic phenotype and the alteration of epigenetic dynamics, the oncometabolites-induced engagement of redox-dependent signaling pathways could contribute both to the neoplastic transformation of healthy cells as well as to the progression of malignancies characterized by germline mutations in SDH and FH and of somatic defects in IDH. These emerging findings reveal a dynamic interaction between the genetic profile, the metabolic status, and the redox tuning of the cell. Moreover, the different impact of oncogenic mutations of the TCA cycle on cellular redox state could contribute to explain the differences in the clinical phenotype and outcome of their associated tumors, opening new perspectives in the comprehension of the molecular mechanisms of oncogenesis and therapeutic targeting of these neoplastic alterations.
